# Precultivation of *Bacillus coagulans* DSM2314 in the presence of furfural decreases inhibitory effects of lignocellulosic by-products during l(+)-lactic acid fermentation

**DOI:** 10.1007/s00253-016-7725-z

**Published:** 2016-07-27

**Authors:** Edwin van der Pol, Jan Springer, Bastienne Vriesendorp, Ruud Weusthuis, Gerrit Eggink

**Affiliations:** 1 Food and Biobased Research, Wageningen University and Research Centre, PO Box 17, 6700 AA Wageningen, The Netherlands; 2 Bioprocess Engineering, Wageningen University and Research Centre, PO Box 16, 6700 AA Wageningen, The Netherlands; 3 Corbion Purac Biochem, PO Box 21, 4200 AA Gorinchem, The Netherlands

**Keywords:** Adaptation, Lignocellulosic by-products, Lactic acid fermentation, *B. coagulans*

## Abstract

By-products resulting from thermo-chemical pretreatment of lignocellulose can inhibit fermentation of lignocellulosic sugars to lactic acid. Furfural is such a by-product, which is formed during acid pretreatment of lignocellulose. pH-controlled fermentations with 1 L starting volume, containing YP medium and a mixture of lignocellulosic by-products, were inoculated with precultures of *Bacillus coagulans* DSM2314 to which 1 g/L furfural was added. The addition of furfural to precultures resulted in an increase in l(+)-lactic acid productivity by a factor 2 to 1.39 g/L/h, an increase in lactic acid production from 54 to 71 g and an increase in conversion yields of sugar to lactic acid from 68 to 88 % *W*/*W* in subsequent fermentations. The improved performance was not caused by furfural consumption or conversion, indicating that the cells acquired a higher tolerance towards this by-product. The improvement coincided with a significant elongation of *B. coagulans* cells. Via RNA-Seq analysis, an upregulation of pathways involved in the synthesis of cell wall components such as bacillosamine, peptidoglycan and spermidine was observed in elongated cells. Furthermore, the gene *SigB* and genes promoted by *SigB*, such as *NhaX* and *YsnF*, were upregulated in the presence of furfural. These genes are involved in stress responses in bacilli.

## Introduction

Lignocellulose is the most abundant biomaterial on earth. It consists for 60–75 % *W*/*W* of sugars, which can be used in fermentation processes to produce biobased chemicals such as lactic acid (van der Pol et al. 2014). Polymerized lactic acid (PLA) can be moulded into bioplastics, which may be a suitable alternative to oil-derived plastics such as polystyrene (PS) and polyethylene (PE) (Garlotta 2001).

Lignocellulosic sugars are polymerized, strongly condensed and covered by lignin, making it difficult for lactic acid-producing bacteria to directly consume these sugars (Fengel and Wegener 1983). A thermo-chemical pretreatment process combined with enzymatic hydrolysis releases the sugars as fermentable monomers or oligomers (Hendriks and Zeeman 2009). However, thermo-chemical pretreatment also leads to the formation of unwanted by-products such as phenolic aldehydes, organic acids and furans, which can inhibit growth and product formation of microorganisms during fermentation processes (Palmqvist and Hahn-Hägerdal 2000a; Palmqvist and Hahn-Hägerdal 2000b; van der Pol et al. 2014). The presence of different by-products in pretreated lignocellulose depends both on the type of thermo-chemical pretreatment used and on the source of lignocellulose used (van der Pol et al. 2014; van der Pol et al. 2015). Furfural is such a by-product, formed by dehydration of xylose during pretreatment at low pH and high temperature. The presence of furfural can generate reactive oxygen species (ROS), which can damage DNA and membranes of microorganisms (Allen et al. 2010; Feron et al. 1991).

*Bacillus coagulans* DSM2314 has been studied as a microbial cell factory for the production of lactic acid (Maas et al. 2008). It is a moderate thermophilic bacterium able to grow in slightly acidic environments. *B. coagulans* can consume both glucose and xylose homofermentatively, reaching a conversion yield of glucose and xylose to lactic acid of over 90 % on a weight basis and reaching a high lactic acid productivity up to 5 g/L/h (Maas et al. 2008). Although *B. coagulans* may be a suitable candidate for the production of lactic acid from lignocellulosic sugars, earlier experiments have shown that *B. coagulans* is relatively sensitive towards lignocellulosic by-products (van der Pol et al. 2016, Walton et al. 2010).

Bacilli like *B. coagulans* are able to adapt to different environmental conditions (Wiegeshoff et al. 2006). Sigma factors play an important role in this adaptation. One of the sigma factors involved in responses towards stress is *SigB*. This gene is upregulated when cells are exposed to stress conditions and tightly regulates expression of around 150 genes (Hecker et al. 2007; Price et al. 2001). Adaptation has not only been observed in environments with suboptimal pH and temperature but has also been observed to play a role in resistance towards toxic compounds (Price et al. 2001).

In this research, we examined whether *B. coagulans* can adapt to environments rich in potentially inhibiting lignocellulosic by-products. Adaptation was accomplished by addition of non-lethal amounts of lignocellulosic by-products to precultures. These precultures were used as inoculum for fermentation processes with medium resembling acid-pretreated sugarcane bagasse, rich in furfural, phenolics and small organic acids.

## Material and methods

### Chemicals

Glucose, xylose and galactose were ordered at Duchefa (The Netherlands) and had a purity of at least 99 % *W*/*W*. Yeast extract, peptone and BIS-Tris were also ordered at Duchefa (The Netherlands). Other chemicals were ordered at Sigma-Aldrich (USA) and had a purity of at least 98 % *W*/*W*, with the exception of formic acid, which was 95 % *W*/*W* pure.

### Microorganism

*B. coagulans* DSM2314 was acquired as freeze-dried stock from the German Collection of Microorganisms and Cell Cultures (DSMZ, Germany). Cells were suspended for 30 min in 5 mL PYPD medium, consisting of 5 g/L yeast extract, 10 g/L peptone, 20 g/L glucose and 10 g/L BIS-Tris, which was pre-sterilized for 20 min at 121 °C. After 30 min of pre-incubation, the cell suspension was transferred to 50-mL anaerobic flasks containing 45 mL fresh PYPD medium, sealed with a rubber cap and incubated without agitation for 16 h at 50 °C to an optical density at 660 nm of around 2. After addition of 15 % *V*/*V* glycerol, cells were stored in 1.5-mL aliquots in cryovials at −80 °C until used.

### Cultivation in anaerobic flasks at 50-mL scale

Cultivation was performed in 50-mL glass anaerobic flasks, sealed with a rubber stopper and aluminium crimp cap, in an incubator set at 50 °C without agitation, starting at a pH of 7.2.

All glass flasks and rubber stoppers, 2× concentrated PYPD medium and milli-Q water were autoclaved at 121 °C for 20 min prior to cultivation, as previously described (van der Pol et al. 2016). Lignocellulosic by-product mixture solutions and single by-product solutions were heated at 85 °C for 1 h. An acid-150 lignocellulosic by-product mixture was used in this experiment, which resembles an acid-pretreated bagasse lignocellulose substrate containing 150 g/L monomeric lignocellulosic sugars (Table 1) (van der Pol et al. 2015). Not only by-product mixtures were tested but also single by-products examined in 50-mL shake flasks. Individual by-products tested were furfural, vanillin, syringaldehyde, acetic acid and formic acid.

**Table 1:** Composition of acid-100 and acid-150 by-product mixtures, as measured after acid pretreatment of bagasse lignocellulose in a previous study (van der Pol et al. 2015). The compositions were used to prepare the model substrate resembling acid-pretreated sugarcane bagasse

Amount (g/L)	Acid-100	Acid-150
Acetic acid	3.13	4.68
Glycolic acid	2.15	3.21
Levulinic acid	2.06	3.08
Formic acid	0.29	0.43
Furfural	1.63	2.44
5-HMF	0.19	0.28
Coumaric acid	0.29	0.43
Ferulic acid	0.04	0.06
4-Hydroxybenzaldehyde	0.04	0.06
Vanillin	0.07	0.10
Syringaldehyde	0.04	0.06

Experiments were performed to observe the effect of different single by-products on the growth of the microorganism and to see whether the microorganism could adapt to these by-products. The initial flasks used in these experiments were inoculated with 250 μL of freezer stock to obtain a starting OD_660_ of 0.01 and contained 25 mL of 2× concentrated PYPD medium, to which either 25 mL milli-Q water (reference) or a 2× concentrated single by-product solution was added. The OD was measured at 660 nm due to expected minimal interference of furfural and the natural brown/yellowish colour of the medium. New flasks, which evaluated adaptation effects, contained 25 mL 2× concentrated PYPD medium and 25 mL of a 2× concentrated single by-product solution. These flasks were inoculated with culture taken from previous anaerobic shake flasks when these reached an OD_660_ of 1, using amounts around 500 μL to obtain a starting OD_660_ in the new flask of 0.01. All experiments were performed in duplicate.

Other experiments were used to observe whether differences occur when the microorganism was grown at different concentrations of furfural. For these experiments, initial flasks were filled with 25 mL 2× concentrated PYPD medium, 0, 0.83, 2.08, 4.17, 8.33 or 12.5 mL of 6 g/L furfural solution and 25, 24.17, 22.92, 20.83, 16.67 or 12.5 mL of milli-Q water, to reach a start concentration of furfural of, respectively, 0, 0.1, 0.25, 0.5, 1 or 1.5 g/L. When the cultures reached an OD_660_ of 1, around 500 μL of culture was transferred to a new flask containing 25 mL of 2× concentrated PYPD medium and 25 mL of either 2× concentrated acid-150 mixture or 6 g/L furfural. The obtained starting OD_660_ in the new flask was 0.01. All experiments were performed in duplicate.

Sterile syringes and needles were used to take samples through the rubber stopper of the anaerobic flasks. Growth was monitored using a spectrophotometer (Ultrospec 2000, Pharmacia Biotech, Sweden) at an optical density wavelength of 660 nm. Furthermore, at different time points, microscope images were taken to follow morphological changes. The microscope (Axioplan, Carl Zeiss, Germany) was equipped with a camera (Axiocam ICc3, Zeiss, Germany), and a Zeiss plan Neofluar 40× lens (Zeiss, Germany) was used.

### Preculture cultivation in 50-mL anaerobic flasks used as inoculum for 1-L fermentations

Fifty-millilitre sterile anaerobic flasks were filled with 25 mL 2× concentrated PYPD medium and 25 mL of either milli-Q water (reference preculture) or 2 g/L furfural dissolved in milli-Q water (furfural-containing preculture), resulting in a final furfural concentration of 1 g/L in the anaerobic flask. The anaerobic flasks were inoculated with 250 μL *B. coagulans* freezer stock to obtain a starting OD at 660 nm of 0.01 and cultivated in an incubator at 50 °C without agitation. When the preculture reached an OD_660_ of 1, which occurs around 15 h for reference precultures and around 24 h for furfural-containing precultures, the preculture was used as inoculum for 1 L pH-controlled fermentations.

Fermentations were performed in 1.5-L Multifors reactors (Infors, Switzerland). Each fermentation started with 100 g/L of sugar mixture, containing of 3.3 g/L galactose, 24.2 g/L xylose and 72.5 g/L glucose, unless stated otherwise. Fermentations performed using peptone medium contain 10 g/L yeast extract and 10 or 20 g/L peptone as nitrogen source (YP10/YP20 medium). Fermentations performed using ammonium medium contain 10 g/L yeast extract, 2 g/L (NH_4_)_2_PO_4_ and 3.5 g/L (NH_4_)_2_SO_4_ as nitrogen source (YA medium). The fermentations using YA medium were performed in duplicate. The medium components were dissolved in 500 mL of milli-Q water in a pre-sterilized fermentation vessel, the pH was set below 5.5 with citric acid to reduce Maillard reactions and the fermenter including 500 mL medium was sterilized for 12 min at 121 °C. After sterilization, 500 mL of either sterile milli-Q water or 500 mL of 2× concentrated by-product mixture, which was pre-heated at 85 °C for 1 h (Table 1), was added to the fermenter. The fermenter was inoculated with 50 mL (5 % *V*/*V*) preculture. The temperature was controlled at 50 °C, the pH was maintained at pH 6 with 4 N KOH and stirring was controlled at 100 RPM. No active aeration was applied during fermentation. At regular intervals during fermentation, samples of 10 mL were taken, the OD at 660 nm was determined and the remainder of sample was frozen at −20 °C for HPLC analysis.

### Analysis of lactic acid, monomeric sugars and furans

Analysis of lactic acid, sugars and other fermentation products of *B. coagulans* which may occur such as ethanol and acetic acid was performed using a Waters e2695 HPLC system (Milford USA) equipped with Waters RI2414 and Waters UV/Vis 2489 (measuring at 210 nm) detectors. The column used was a Shodex RS pak KC-811 ion exchange column (length 300 mm, I.D. 8 mm), controlled at 65 °C. As eluent, 3 mM H_2_SO_4_ in milli-Q water was used. The flow used was 1 mL/min. Samples obtained during fermentation were de-frozen prior to analysis. Two hundred fifty microlitres of this sample was mixed with 250 μL of internal standard, containing 0.25 g/L phthalic acid and 500 μL of milli-Q water. Samples were filtered using 0.2 μm Spartan filters, and supernatants were measured using HPLC.

To determine furan concentrations, UPLC-MS/MS measurements were performed using a Dionex Ultimate 3000 RSLC system, equipped with a Waters Acquity BEH C18 RP column, in combination with a Thermo Scientific^TM^ LCQ Fleet Ion Trap Mass Spectrometer, as previously described (van der Pol et al. 2015).

### Calculations to determine amount of lactic acid formed per batch based on KOH added

Due to a large increase in volume during fermentation, values for lactic acid produced were recalculated to gram per batch instead of grams per litre, used to determine yields and total lactic acid produced. Volume of the reactor at time point *t* (*V*_R,*t*_) was calculated based on the amount of base added as fraction of the total amount of base added at time point *t* (*B*_*t*_/*B*_end_), volumes of the reactor at *T* = 0 in *L* (*V*_F,0_), determined volume of the reactor at the end of the SSF in litre (*V*_F,end_) and the total volume of sample taken at time point *t* in litre (*V*_S_). The amount of lactic acid in gram at time point *t* (*A*_LA,*t*_) was calculated based on the concentration of lactic acid in grams per litre determined via HPLC at time point *t* (*C*_LA,*t*_), the volume of the reactor at time point *t* (*V*_R,*t*_) and a correction factor for the amount of lactic acid taken out by sampling at sample *n* (CF_LA,*n*_).

*A*_LA,*t*_ = *C*_LA,*t*_ × *V*_R,*t*_ + CF_LA,*n*_ with ${V}_{\mathrm{R},t}={V}_{\mathrm{F},0}+\frac{B_t}{B_{\mathrm{end}}}\times \left({V}_{\mathrm{F},\mathrm{end}}-{V}_{\mathrm{F},0}\right)-{V}_{\mathrm{S}}$ and ${\mathrm{CF}}_{\mathrm{LA},n}={\displaystyle \sum_{i=1}^{n-1}{C}_{\mathrm{LA},i}\times {V}_{S,i}}$

Lactic acid production can be estimated based on the amount of KOH added to the reactor. Lactic acid concentration at a certain time (*A*_LA,*t*_) was calculated by taking the amount of base added at a certain time point (*B*_*t*_) logged in real-time by the Infors control unit of the fermentor, divided by the total amount of base added (*B*_*t* = end_) and multiplied by the final lactic acid titre (*A*_LA,*t* = end_) measured in triplicate via HPLC. The overall calculation is:

$$ {A}_{\mathrm{LA},t}=\frac{B_t}{B_{t\kern0.5em =\kern0.5em \mathrm{end}}}\times {A}_{\mathrm{LA},t\kern0.5em =\kern0.5em \mathrm{end}} $$

No difference in lactic acid production larger than 2 g was observed between the calculations based on the KOH addition and HPLC analysis of samples taken at different time points during the fermentations.

### Isolation of RNA and DNA

Four reference and four furfural-containing cultures were prepared in 50-mL anaerobic flasks as described previously for precultivation. When the cultures reached an OD of 1, the cultures were transferred to 50-mL Greiner tubes and directly frozen using liquid nitrogen. After overnight storage at −80 °C, samples were thawed and vortexed for 5 s. Ten-milliliter sample of each culture was placed in 15-mL Greiner tubes, together with one extra sample from a reference culture for DNA extraction. The nine tubes were centrifuged at 4 °C for 15 min at 4700 RPM. After centrifugation, the tubes were kept on ice whenever possible. Supernatant was removed, cells were resuspended in 0.5 mL ice cold TE buffer (pH 8) and transferred to 2-mL tubes containing specialized Lysing matrix E beads (Lysing matrix E, MP Biomedicals, Ohio, USA). After addition of 500 μL extraction buffer (0.6 % SDS and 0.2 M sodium acetate (pH 5.2)), samples were vigorously shaken using a Precellys 24 homogenizer (Bertin Technology, France) for 2× 30 s at 5500 RPM, with a 60-s interval. Five hundred microlitres of phenol-choloroform (50–50 % *V*/*V*) was added, tubes were vortexed for 5 s and kept for 10 min on ice. Tubes were then centrifuged for 5 min at 10,000 rpm and 4 °C. Four hundred microlitres of the liquid fraction was transferred to a fresh RNA-free Eppendorf vial, 400 μL of chloroform was added and samples were centrifuged for 3 min at 12,000 RPM and 4 °C.

After washing with chloroform, either a RNA or DNA kit was used for the purification RNA and gDNA, respectively. For RNA, 300 μL of the water phase was transferred to a clean Eppendorf vial, and 300 μL of lysis buffer was added from an RNA extraction kit. The protocol of the extraction kit (Roche High Pure RNA isolation kit v12, Roche, Switzerland) was followed, with the exception that a DNAse incubation time of 45 min was used. For DNA purification, a Sigma-Aldrich Bacterial gDNA kit was used according to protocol (NA2110-1KT, Sigma-Aldrich, USA). The extraction resulted in four purified RNA samples containing biological replicates of the reference culture, four purified RNA samples containing biological replicates of the furfural-containing culture and one purified gDNA sample.

### DNA analysis and assembly

gDNA quantities in the purified gDNA sample was estimated using a Nanodrop Lite system (Thermo Scientific, DE, USA). The integrity of the DNA was observed using gel electrophoresis. Samples were sent to Baseclear (The Netherlands), which sequenced the gDNA via paired-end sequencing using an Illumina HiSeq 2500 system. The obtained FASTQ file was filtered for reads containing adapters and/or PhiX control signals and for reads that did not pass the Illumina Chastity filtering. A second filtering was performed using FASTQC quality control tool version 0.10.0.

For DNA assembly, the raw Illumina data, containing paired ends of 126 bp reads, was used as input for de novo assembly via IDBA (idba-1.1.1, Peng et al. 2012) using a K-mer range from 20 to 120, with steps of 3, and via Ray (Ray 2.3.1, Boisvert et al. 2010) with K-mers ranging from 15 to 81, selecting for low scaffold runs. Both runs gave comparable results, whilst the output from Ray with a K-mer of 61 was chosen for final assembly, since it contained less contigs, and protein prediction was of slightly higher quality compared to IDBA run. The final assembly contained 3,569,489 bp and a GC content of 46.3 %. Genes were annotated using PROKKA v1.11 (Seemann 2014) which predicted a total of 3402 coding sequences, 11 rRNAs and 60 tRNAs,

### RNA-seq analysis and assembly

RNA quantities in the purified RNA samples were estimated using a Nanodrop Lite system (Thermo Scientific, DE, USA). The integrity of the RNA was observed using gel electrophoresis. Samples were sent to Baseclear (The Netherlands), which analysed the RNA samples using deep sequencing on an Illumina HiSeq 2500 system. This resulted in raw sequence data containing single-end reads of 50 bp. A FASTQ file was generated using Illumina Casava v1.8.3. This file was filtered by Illumina chastity filtering, removal of adapters and quality assessment based in FASTQC quality control tool v0.10.0.

RNA sequences were mapped to the reference sequence using Burrows-Wheeler Aligner (BWA) (Li 2013) run with default settings. RNA-seq analysis was performed using DESeq method (Mortazavi et al. 2008) and was used to compute differential expression and estimate *p* values (Anders and Huber 2010). *P* values were adjusted for multiple testing with Benjamini and Hochberg (1995) approach for adjusting the false discovery rate (FDR). A cutoff *p* value of 0.05 was used.

The raw Fastq database, containing single-end 50 bp reads, has been created with Illumina Casava and has been uploaded to the NCBI sequence read archive (SRA) under project number SRP075359/bioproject PRJNA320675.

## Results

### Effect of precultivation in presence of lignocellulosic by-products on growth of *B. coagulans* in 50-mL anaerobic flasks

**Fig. 1:**
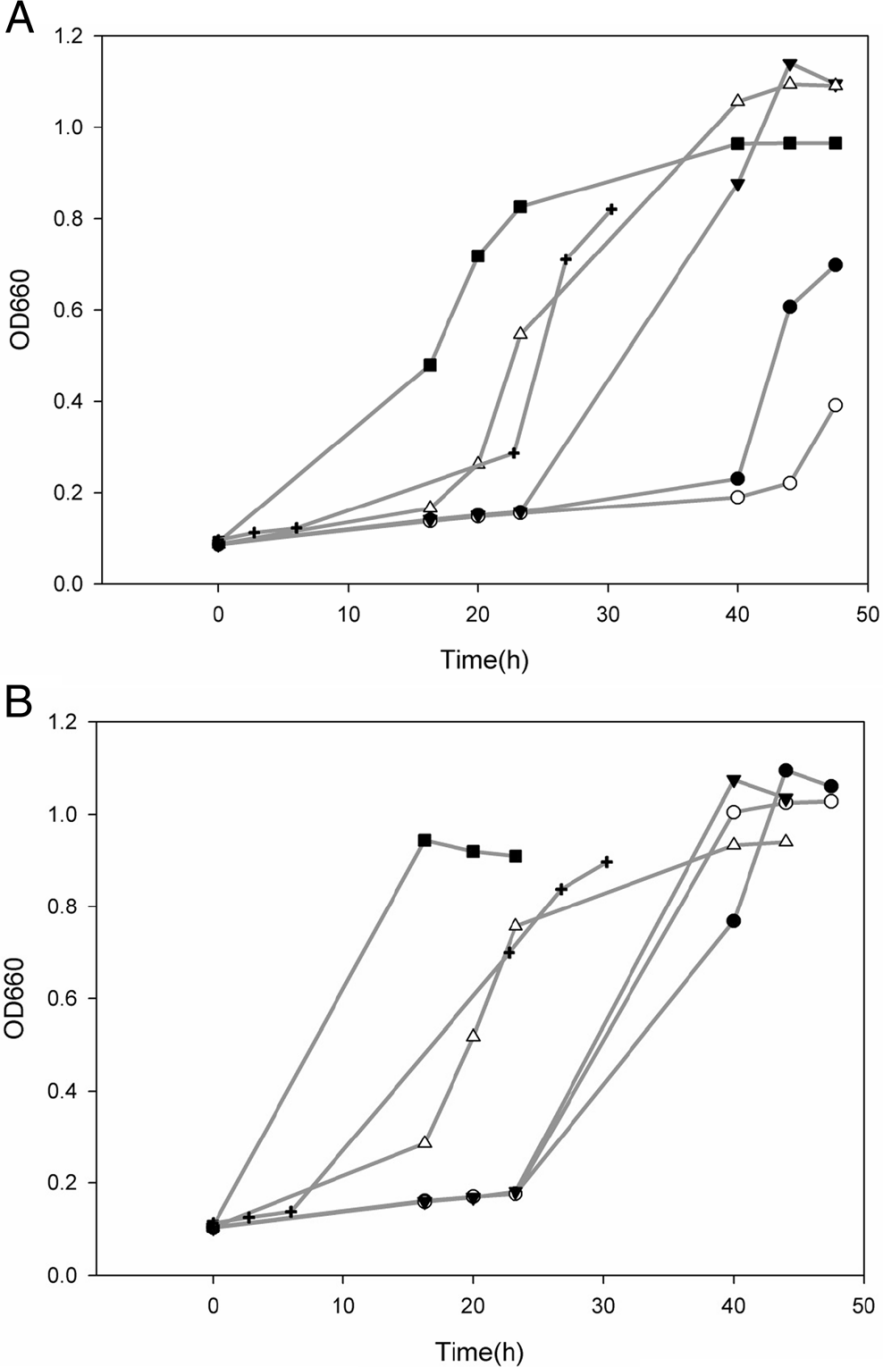
Effect of furfural concentration during adaptation on growth of *B. coagulans* in 50-mL anaerobic flasks containing **a** 3 g/L furfural and **b** acid-150 mixture of by-products. All experiments were performed in duplicate, and average results are shown. The following furfural concentrations were tested during precultivation: *black circle*, 0 g/L; *white circle*, 0.1 g/L; *black down-pointing triangle*, 0.25 g/L; *white up-pointing triangle*, 0.5 g/L; *black square*, 1 g/L; *plus sign*, 1.5 g/L

**Fig. 2:**
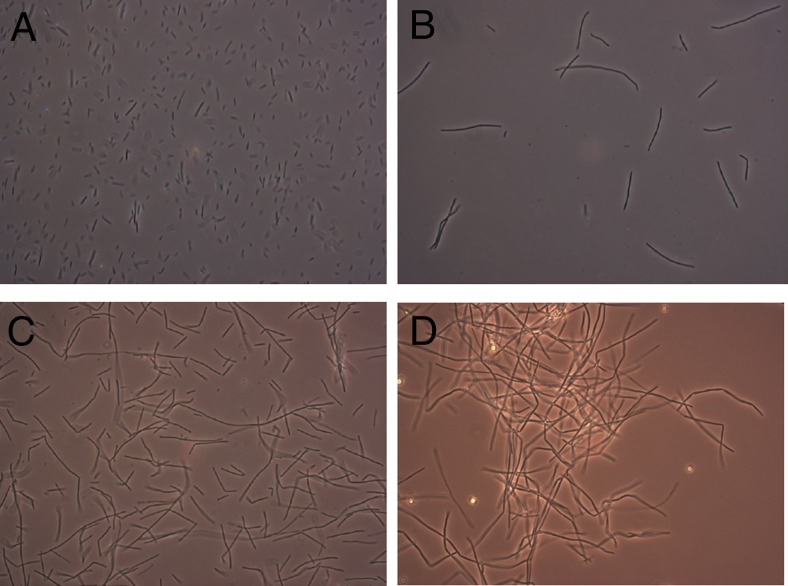
Effect of growth in the presence of by-products on the morphology of *B. coagulans*. **a** Reference preculture, no by-products present. **b** Preculture containing 1 g/L furfural in late-exponential growth phase. **c** YA medium containing acid-100 mixture cultivated for 24 h, inoculated with furfural-containing preculture. **d** YP20 medium containing acid-150 mixture cultivated for 40 h, inoculated with furfural-containing preculture

*B. coagulans* was precultivated in the presence of either organic acids (formic acid, acetic acid), phenolic aldehydes (vanillin, syringaldehyde) or furfural. These precultures were used to inoculate 50-mL anaerobic flasks containing the same by-products (Table 2). Precultivation in the presence of organic acids increased fermentation time required to reach maximum OD in the subsequent fermentation by 20–40 %. Precultivation of *B. coagulans* in the presence of vanillin did not have an effect on fermentation time. The presence of syringaldehyde resulted in a small reduction of fermentation time by 6 h (a reduction of 23 %) (Table 2). When the cells were precultivated in the presence of furfural, a reduction of approximately 50 % in fermentation time was observed, mainly due to a reduction in lag phase (Fig. 1). HPLC analysis showed that by-product concentrations did not change significantly during the anaerobic flask experiments (data not shown), indicating that they were not consumed or converted by *B. coagulans*. Cells grown in the presence of furfural showed a change in morphology: the cell length increased drastically (Fig. 2). When these cells were used as inoculum for a new 50-mL flask which did not contain by-products, cell morphology changed to a non-elongated state within seven generations, a morphology similar to what is observed in a reference preculture.

**Table 2:** Effect of the presence of lignocellulosic by-products during precultivation on growth of *B. coagulans* in presence of the same by-product

	By-product concentration during precultivation	By-product concentration during cultivation	Batch time of cultivation
	g/L	g/L	h
Reference	0	0	16
Furfural	0	2	40
	1	2	23
	2	2	25
	0	3	60
	1	3	27
	2	3	32
Vanillin	0	4	20
	2	4	19
	3	4	20
Syringaldehyde	0	5	26
	2.5	5	20
Acetic acid	0	20	23
	10	20	28
Formic acid	0	7.5	16
	7.5	7.5	23

Batch time of cultivation was the time required to reach maximum OD

To optimize adaptation, different furfural concentrations were tested during precultivation. These precultures were used as inocula for flasks containing either 3 g/L furfural or acid-150 mixture of by-products (Table 1). For both situations, it was found that fastest growth occurred when 1 g/L furfural was added to the preculture (Fig. 1). Therefore, a furfural concentration of 1 g/L was used during precultivation in the next experiments.

Although sufficient substrate was still available at the end of these fermentations, the reduction of pH due to the production of lactic acid stopped growth in the anaerobic flasks. To accurately assess lactic acid productivity, yield and titre, pH-controlled fermenter experiments were performed.

### Studies in pH-controlled fermentations

pH-controlled fermenters with a start volume of 1 L were either inoculated with a furfural-containing preculture or a reference preculture to which no by-products were added. Two different media were used. The first medium was similar to the medium used at 50-mL scale and contained 100 g/L sugars (72.6 % glucose, 24.2 % xylose, 3.2 % galactose), 10 g/L yeast extract and 10 or 20 g/L peptone (YP10/YP20 medium). In the second medium, peptone was replaced by ammonium salts (YA medium).

**Fig. 3:**
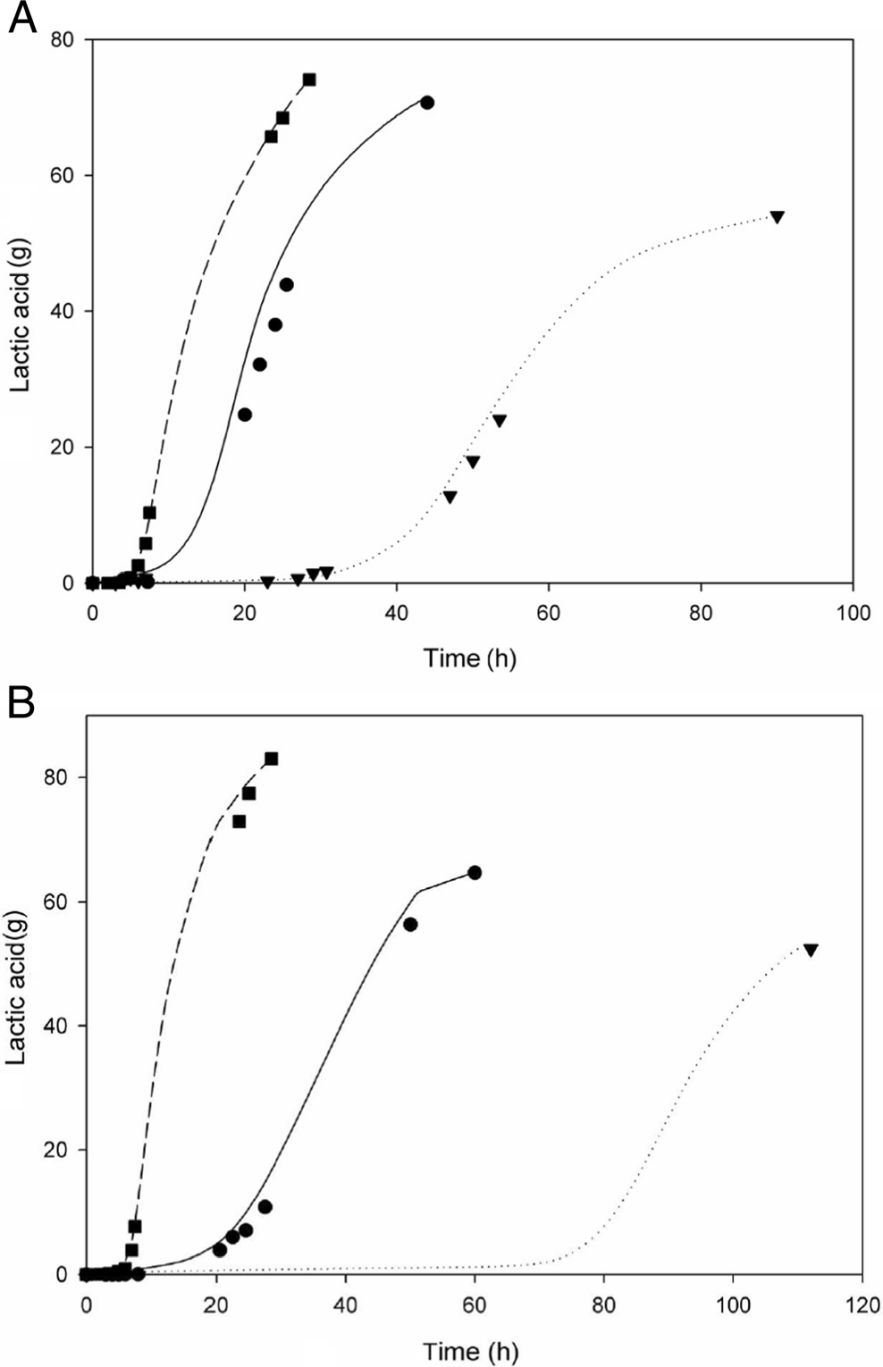
Effect of adaptation to furfural during precultivation on lactic acid production by *B. coagulans* growing in the presence of a mixture of by-products using YP medium. Lactic acid production was measured by HPLC (*symbols*) or calculated based on KOH addition (*lines*). **a** YP10 medium and acid-100 by-product mixture. **b** YP20 medium and acid-150 by-product mixture. *Black square and broke line*: Fermentation not containing by-products, inoculated with reference preculture. *Black circle and straight line*: Fermentation containing by-products, inoculated with furfural-containing preculture. *Black down-pointing triangle and dotted line*: Fermentation containing by-products, inoculated with reference preculture

Addition of furfural to precultures, used as inocula in fermenters containing YP10 medium and acid-100 mixture of by-products (Table 1), reduced the total fermentation time by 50 % (Table 3 and Fig. 3a). This resulted in an increase in average lactic acid productivity from 0.56 to 1.39 g/L/h. Furthermore, total lactic acid production increased from 54.0 to 71.5 g.

**Table 3:** Effect of precultivation on pH-controlled fermentations with a start volume of 1 L using different media, with or without the addition of acid-100/acid-150 by-product mixture

Preculture	By-products	*C*_s_ (g/L)	Medium	*C*_La_ (g/L)	*A*_La_ (g)	*Q*_v,av_ (g/L/h)	*Q*_v,max_ (g/L/h)	*Y*_s/La_	Time (h)
Reference	None	100	YP10	52.9	74.2	2.23	4.6	90 %	29
Furfural	Acid-100	100	YP10	50.4	71.5	1.39	3.0	88 %	44
Reference	Acid-100	100	YP10	40.5	54.0	0.56	1.4	68 %	85
Reference	None	100	YP20	59.3	83.0	2.50	5.1	92 %	29
Furfural	Acid-150	100	YP20	46.6	65.3	0.93	1.6	81 %	60
Reference	Acid-150	100	YP20	39.6	52.4	0.42	1.2	68 %	110
Reference	None	100	YA	55.6	78.6	2.40	3.90	86 %	28
Furfural	Acid-100	100	YA	47.4	68.4	2.06	2.96	84 %	28
Reference	Acid-100	100	YA	47.3	66.4	0.81	1.43	76 %	70
Furfural	Acid-100	125	YA	70.8	103.9	1.68	3.80	91 %	52

As inoculum, 5 % *V*/*V B. coagulans* culture was used, either precultivated in the presence of 1 g/L furfural (furfural preculture) or in the absence of furfural (reference preculture)

*C*_*s*_ concentration of sugar at the start of the fermentation, 100 g/L is a mix of 72.5 % glucose, 24.2 % xylose and 3.3 % galactose, whilst medium with 125 g/L sugars is solely glucose; *C*_*La*_ concentration of lactic acid at the end of the fermentation in grams per litre; *A*_*La*_ total lactic acid produced in gram; *Q*_*v,av*_ average volumetric lactic acid productivity in grams per litre per hour; *Q*_*v,max*_ maximum volumetric lactic acid productivity in grams per litre per hour; *Y*_*S/La*_ conversion yield of consumed sugars to lactic acid in weight per weight; *Time* total fermentation time, from inoculation to reaching final lactic acid concentration

A similar result was obtained in fermenters containing YP20 medium and acid-150 by-product mixture. Addition of furfural to precultures increased the lactic acid productivity from 0.42 to 0.93 g/L/h and the yield from 68 % (*W*/*W*) to 81 % (*W*/*W*). The total amount of lactic acid produced increased by 25 % to 65.3 g (Table 3 and Fig. 3b).

The morphological changes that occurred in anaerobic flasks in the presence of furfural were also observed in these fermenter experiments: In the late-exponential phase of the fermentation, all cells were strongly elongated (Fig. 2).

**Fig. 4:**
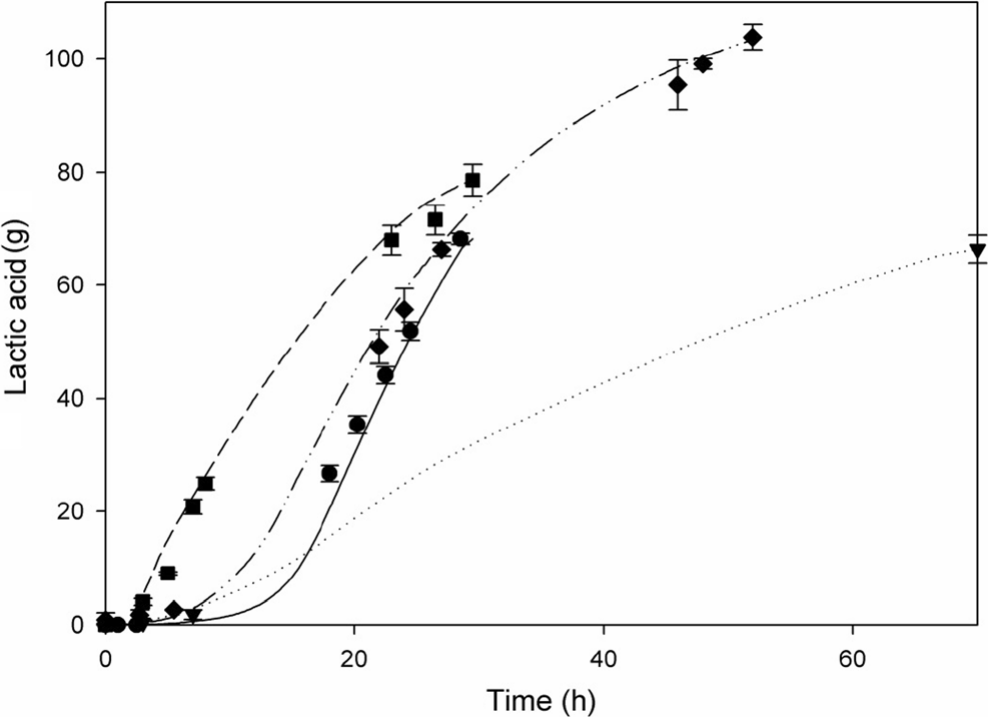
Effect of adaptation to furfural during precultivation on lactic acid production by *B. coagulans* growing in the presence of a mixture of by-products, using a YA medium. Lactic acid production was measured via HPLC (*symbols*) or calculated based on KOH addition (*lines*). Fermentations were performed in duplicate; *error bars* represent deviation from the average lactic acid amount measured. *Black square* and *broken line*: Fermentation not containing by-products, inoculated with reference preculture, 100 g/L mixed sugars. *Black circle* and *straight line*: Fermentation containing by-products, inoculated with furfural-containing preculture, 100 g/L mixed sugars. *Black down-pointing triangle and dotted line*: Fermentation containing by-products, inoculated with reference preculture, 100 g/L mixed sugars. *Black diamond* and *straight dotted line*: Fermentation containing by-products, inoculated with furfural-containing preculture, 125 g/L glucose

Using furfural-containing inocula instead of reference inocula in fermenters containing YA medium and acid-100 by-product mixture did not result in an increased lactic acid titre (Table 3 and Fig. 4). However, the total fermentation time was reduced by 60 %, resulting in an increase in lactic acid productivity from 0.8 to 2.1 g/L/h.

**Fig. 5:**
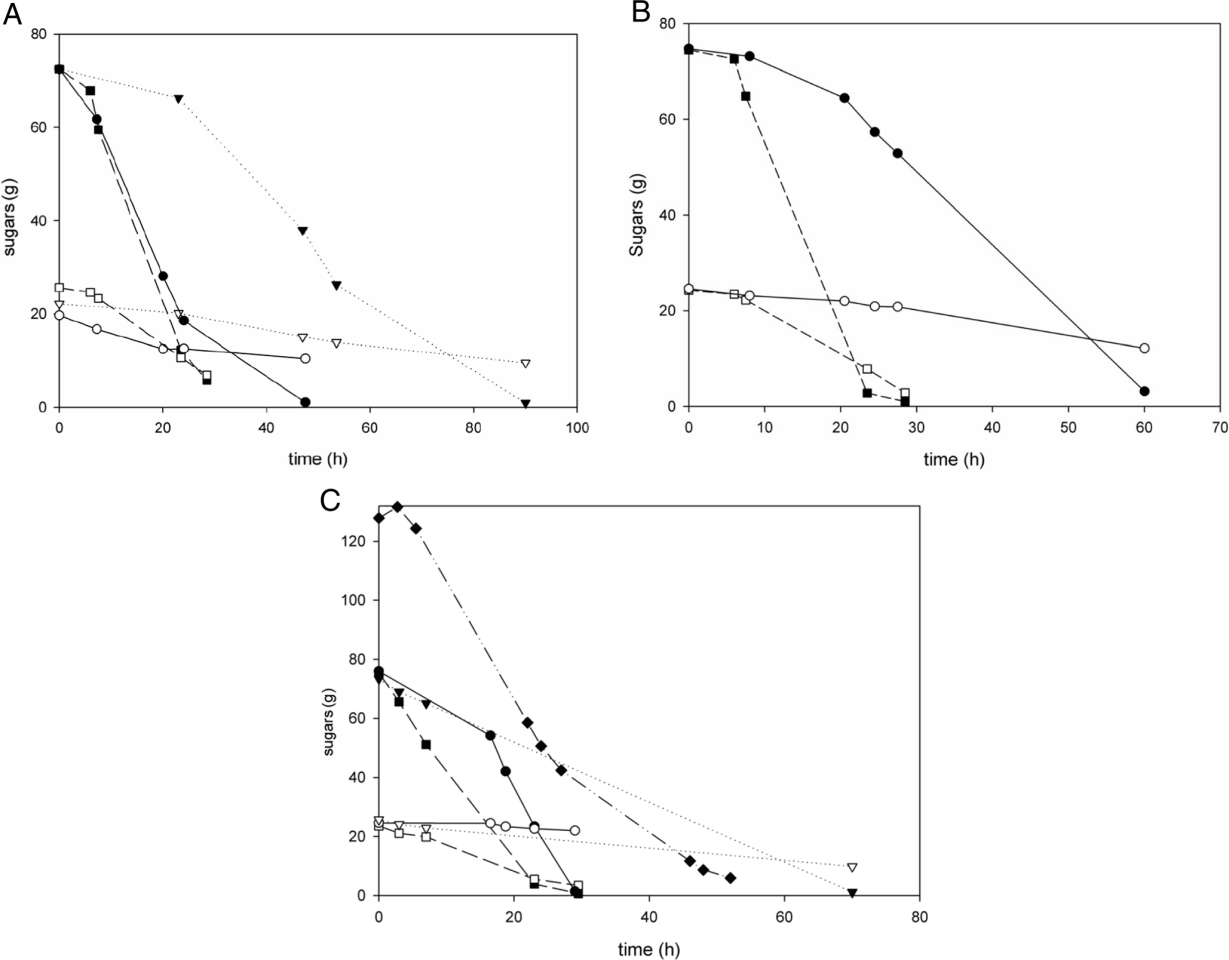
Glucose (*closed symbols*) and xylose (*open symbols*) amounts present in 1 L controlled fermentations. The following media were used: **a** YP10 medium, **b** YP20 medium and **c** YA medium. *Black square* and *broken line*: Fermentation not containing by-products, inoculated with reference preculture. *Black circle* and *straight line*: Fermentation, inoculated with furfural-containing preculture, containing by-product mixture acid-100 (**a**, **c**) or acid-150 (**b**). *Black down-pointing triangle and dotted line*: Fermentation, inoculated with reference preculture, containing by-product mixture acid-100 (**a**, **c**) or acid-150 (**b**). *Black diamond* and *straight dotted line*: Fermentation, inoculated with furfural-containing preculture, containing 125 g/L glucose as sole carbon source and by-product mixture acid 100 (**c**)

In fermentations containing lignocellulosic by-products, xylose was only partially consumed (Fig. 5). At the start of the fermentation, 24.2 g of xylose was present. In both reference fermentations using YA medium and reference fermentations using YP20 medium, a final residual xylose amount of around 3 g was measured. On the other hand, a final residual xylose amount of 21 g was observed in acid-100 containing fermentation processes on YA medium, and 16.6 g of xylose was residual when YP10 medium was used. Addition of furfural to the preculture did not influence the total xylose consumption in the subsequent fermentation process.

The lactic acid production potential on glucose was determined for *B. coagulans*, pregrown in the presence of furfural, in YA medium, containing 125 g of glucose as carbon source (Table 3 and Fig. 4). These fermentations resulted in a total lactic acid production of 103 g in 52 h with an average productivity of 1.68 g/L/h and a conversion yield of glucose to lactic acid of 91 % *W*/*W*.

### RNA-seq analysis of *B. coagulans* grown in the presence or absence of furfural

RNA-seq analysis was performed to identify possible mechanisms used by the microorganism to reduce the toxicity of by-products when the cells are precultivated in the presence of furfural. mRNA expression levels in cultures grown in the presence and absence of furfural, with cultivation conditions similar to precultivation, were compared (Table 4). Surprisingly, genes involved in detoxification of ROS were downregulated threefold in the presence of furfural. Peptidoglycans and spermidine are part of the cell wall structure of *Bacillus* strains. The pathway leading from peptidoglycan precursor UDP-*N*-acetylglucosamine to more complicated peptidoglycan structures was upregulated by a factor 1.4–2.3 when furfural was present. The pathway also requires the presence of UDP-GlcNAc, another precursor necessary for the production of peptidoglycans. Genes involved in UDP-GlcNAc production were upregulated by 1.3–3.2-fold. Other genes involved in the production of more complicated peptidoglycans such as UDP-*N*-acetylgalactosamine-undecaprenyl-phosphate *N*-acetylgalactosaminephosphotransferase were upregulated 3.2–5.3-fold. Genes involved in spermidine production, another molecule which can be part of the cell wall, were upregulated by 2.2–4.4-fold, whilst genes involved in the conversion of glutamate to ornithine, a precursor of spermidine, were upregulated 2.8–13.5-fold, and genes in the pathway leading from ornithine to proline were downregulated 2-fold.

**Table 4:** Differences in gene expression between reference precultures and furfural precultures of *B. coagulans*, determined using RNA-Seq analysis

	Gene name	Enzyme classification	Amount of RNA measured		Fold change	*P* value
			Control	furfural		
Uracil synthesis	Carbamoyl-phosphate synthase (large chain)	6.3.5.5	6947 ± 1543	172 ± 19	0.03	<0.001
	Carbamoyl-phosphate synthase (small chain)	6.3.5.5	1419 ± 223	47 ± 9	0.03	<0.001
	Aspartate carbamoyltransferase	2.1.3.2	735 ± 134	43 ± 11	0.06	<0.001
	Dihydroorotase	3.5.2.3	1412 ± 207	45 ± 8	0.03	<0.001
	Dihydroorotate dehydrogenase B	1.3.1.14	1435 ± 292	58 ± 11	0.04	<0.001
	Orotate phosphoribosyltransferase	2.4.2.10	865 ± 215	19 ± 5	0.02	<0.001
	Orotidine 5′-phosphate decarboxylase	4.1.1.23	1443 ± 156	30 ± 5	0.02	<0.001
Alternative UDP production from cytosine	Cytosine permease		692 ± 87	6832 ± 1424	9.9	<0.001
	Pyrimidine-nucleoside phosphorylase	2.4.2.2	3118 ± 81	6193 ± 485	2.0	<0.001
	Cytidine deaminase	3.5.4.5	1150 ± 82	1911 ± 106	1.7	<0.001
	Uracil phosphoribosyltransferase	2.4.2.9	5123 ± 888	11,996 ± 2241	2.3	<0.001
	Uridylate kinase	2.7.4.22	3877 ± 426	5703 ± 543	1.5	<0.001
Bacillosamine production	UDP-*N*-acetylglucosamine 1-carboxyvinyltransferase 1	2.5.1.7	52,809 ± 5883	123,575 ± 11,825	2.3	<0.001
	d-Alanyl-d-alanine carboxypeptidase DacA	3.4.16.4	18,418 ± 2280	42,332 ± 4796	2.3	<0.001
Production of UDP-GlcNAc	Glutamine-fructose-6-phosphate aminotransferase	2.6.1.16	27,672 ± 4657	87,570 ± 14,507	3.2	<0.001
	Bifunctional protein GlmU	2.7.7.23	25,201 ± 2539	54,997 ± 4154	2.2	<0.001
Other genes involved in cell wall peptidoglucan production	UDP-4-amino-4-deoxy-l-arabinose-oxoglutarate aminotransferase	2.6.1.87	2258 ± 156	7185 ± 2911	3.2	<0.001
	UDP-*N*-acetylgalactosamine-undecaprenyl-phosphate *N*-acetylgalactosaminephosphotransferase	2.7.8.40	565 ± 124	2467 ± 1134	4.4	<0.001
	UDP-*N*-acetyl-alpha-d-glucosamine C6 dehydratase	4.2.1.135	2194 ± 275	11,681 ± 4131	5.3	<0.001
Spermidine synthesis and transportation	Carboxynorspermidine synthase	1.5.1.43	3390 ± 507	14,834 ± 2472	4.4	<0.001
	Carboxynorspermidine/carboxyspermidine decarboxylase	4.1.1.96	3852 ± 659	12,476 ± 1888	3.2	<0.001
	Spermidine/putrescine transport system permease protein PotB		593 ± 39	1145 ± 272	2.0	0.07
	Spermidine/putrescine import ATP-binding protein PotA	3.6.3.31	1296 ± 80	2576 ± 589	2.0	<0.001
	Spermidine/putrescine-binding periplasmic protein precursor		1159 ± 75	2367 ± 587	2.0	<0.001
Production of precursors for spermidine production such as ornithine	Arginine biosynthesis bifunctional protein ArgJ	2.3.1.35	233 ± 26	818 ± 112	3.5	<0.001
	Acetylglutamate kinase	2.7.2.8	80 ± 25	232 ± 67	2.9	0.005
	*N*-acetyl-gamma-glutamyl-phosphate reductase	1.2.1.38	239 ± 32	814 ± 134	3.4	<0.001
	Acetylornithine aminotransferase	2.6.1.11	118 ± 78	295 ± 74	2.5	0.006
	Ornithine aminotransferase	2.6.1.13	354 ± 161	4785 ± 639	13.5	<0.001
	Pyrroline-5-carboxylate reductase	1.5.1.2	2536 ± 530	1250 ± 90	0.49	<0.001
	Gamma-glutamyl phosphate reductase	1.2.1.41	3646 ± 492	1494 ± 194	0.54	<0.001
	1-Pyrroline-5-carboxylate dehydrogenase	1.2.1.88	165 ± 83	79 ± 17	0.48	0.04
Entner-Doudoroff pathway	Altronate oxidoreductase	1.1.1.58	459 ± 101	3453 ± 294	7.5	<0.001
	Altronate dehydratase	4.2.1.7	241 ± 173	1428 ± 144	5.9	<0.001
	2-Dehydro-3-deoxygluconokinase	2.7.1.45	953 ± 128	3422 ± 735	3.6	0.07
	KHG/KDPG aldolase	4.1.3.16	176 ± 49	733 ± 60	4.2	<0.001
Involved in degradation of reactive oxygen species (ROS)	Superoxide dismutase (Mn)	1.15.1.1	49,363 ± 7333	13,805 ± 2269	0.28	<0.001
	Thioredoxin reductase	1.8.1.9	30,512 ± 3734	9465 ± 1700	0.31	<0.001
	Catalase	1.11.1.6	80,961 ± 15,194	26,199 ± 8907	0.32	<0.001
Synthesis of pantoate	3-Methyl-2-oxobutanoate hydroxymethyltransferase	2.1.2.11	5473 ± 1011	818 ± 35	0.15	<0.001
	Pantothenate synthetase	6.3.2.1	3896 ± 444	745 ± 63	0.19	<0.001
Sigma factor F	RNA polymerase sigma F factor		462 ± 26	1797 ± 363	3.9	<0.001
	Anti-sigma F factor antagonist		207 ± 17	813 ± 143	3.9	<0.001
	Anti-sigma F factor		267 ± 17	1076 ± 143	4.0	<0.001
Sigma factor B	Anti-sigma-B factor antagonist		1242 ± 64	2344 ± 260	1.9	<0.001
	RNA polymerase sigma-B factor		2014 ± 192	4941 ± 920	2.5	<0.001
Sigma factor B-regulated stress proteins	Stress response protein YsnF		346 ± 38	1296 ± 283	3.7	<0.001
	Stress response protein NhaX		190 ± 23	759 ± 132	4.0	<0.001
	General stress protein 13 (Yugl)		15,960 ± 1782	23,581 ± 2314	1.5	<0.001
	Stress response protein CsbD		201 ± 97	396 ± 76	2	0.015
	General stress protein 20U (DPS)		2576 ± 285	6187 ± 1470	2.4	<0.001
	General stress protein 17M (YflT)		843 ± 124	2038 ± 272	2.4	<0.001
	General stress protein 30 (YxaB)		523 ± 69	1939 ± 190	3.7	<0.001
Pathway from pyruvate to formate and acetyl-CoA	Formate acetyltransferase	2.3.1.54	4916 ± 664	32,408 ± 3242	6.6	<0.001
	Pyruvate formate-lyase-activating enzyme	1.97.1.4	1034 ± 142	6914 ± 731	6.7	<0.001
Sigma A	RNA polymerase sigma factor SigA		16,055 ± 1485	14,655 ± 779	0.91	0.37

A fold change above 1 relates to a higher expression of the gene in the furfural preculture, whilst a fold change below 1 relates to a higher expression of the gene in the reference preculture. The *p* value expresses the significance of the fold change

Most genes in *B. coagulans* are tightly regulated by sigma factors (Price et al. 2001, Schmidt et al. 1990). Two sigma factors were observed to be upregulated in the presence of furfural, namely *SigF* by 4-fold and *SigB* by 2.5-fold. For both *SigF* and *SigB*, anti-sigma factor antagonists, required for the activation of the sigma factors, were upregulated in the presence of furfural. *SigB* controls the expression of other genes such as *YsnF* and *NhaX*, which were found to be upregulated by 3.7–4.0-fold.

The pyrimidine synthesis pathway, leading from aspartate and carbomoyl phosphate to UDP, was downregulated by 20- to 50-fold in the presence of furfural, with actual expression values close to zero. An alternative in *B. coagulans* for the synthesis of UMP/UDP is forming them from compounds present in the rich YP medium used during cultivation. A 1.8–2.5-fold upregulation was observed for genes involved in the conversion of cytosine to UMP via cytidine and from uracil to UMP, whilst a transporter involved in cytosine uptake was found to be upregulated 10-fold.

## Discussion

Addition of furfural during precultivation of *B. coagulans* DSM2314 results in an improved fermentation on substrates rich in lignocellulosic by-products. This effect has to our knowledge not been described before. Addition of other by-products during precultivation had no or a less significant impact.

This improvement was observed in lactic acid yield, titres and productivity, both on YP media and YA media. On YP medium, also a strong reduction of the lag phase was also observed, whereas the reason for this difference is unknown. Elongation of cells, which appeared during precultivation in the presence of furfural, seemed to be related to the improvement of fermentation using by-product-rich substrates. This elongation was reversed when cells were transferred back to original medium, suggesting that the effects of precultivation are adaptation effects and not based on changes in the genotype.

In the presence of acidic by-products, elongation of the cells was not observed, whilst adaptation in the presence of acidic by-products was also not observed (Table 2). This may further confirm the relation between elongation and improvement of fermentation in by-product-rich substrates. In the presence of syringaldehyde and vanillin, elongation was observed, however not at the same level as observed in presence of furfural.

When reference precultures were used in by-product rich medium, the adaptation may also occur during the long lag phase. This adaptation most likely occurs since furfural was also present in the acid-100 and acid-150 mixture (Table 1). However, since the fermentation medium also contains numerous other by-products, the adaptation takes 30 h (acid-100) to 60 h (acid-150) to occur. By allowing the cells to adapt during preculture, the adaptation can be accelerated. It was observed that the amount of furfural added had a significant impact on the performance during the actual fermentation (Fig. 1). Furthermore, it was observed that when the adaptation already occurred during precultivation, it also had a positive effect on yield and maximum productivity.

Conversion of xylose to lactic acid was decreased in the presence of by-products, resulting in a high residual xylose concentration at the end of fermentation (Fig. 5). In reference fermentations, when cells switched from co-consumption of glucose and xylose to consumption of xylose as sole carbon source, lactic acid production immediately decreased from 1.9 to 1.2 g/L/h. It may be speculated that capacity of pathways involved in xylose fermentation is too low to produce the amount of energy required for the high maintenance in media rich in by-products.

Ninety-four percent of the sugars present in acid-pretreated sugarcane bagasse fibres after enzymatic hydrolysis is glucose, whilst most hemicellulose sugars such as xylose are present in the liquid fraction (van der Pol et al. 2015). *B. coagulans* precultivated in the presence of furfural can be used to efficiently produce lactic acid from glucose present in bagasse fibres, as shown in Fig. 4. However, fermentation of the liquid fraction containing mostly xylose may not be efficient. A possible solution is to ferment this fraction in a separate process by a different microorganism.

Microorganisms are able to reduce furfural toxicity by either converting the furfural or by increasing its tolerance to this inhibitor. *Escherichia coli*, *Cupriavidus basilensis* and *Saccharomyces cerevisiae* were previously shown to be able to convert furfural, either into furfuryl alcohol (Gutiérrez et al. 2002; Laadan et al. 2008) or into 2-furoic acid/2,5-furandicarboxylic acid (Koopman et al. 2010; Nichols et al. 2008), compounds that are less toxic for these microorganisms. No changes in furfural concentration or formation of furfuryl alcohol or furoic acid was observed during precultivation of *B. coagulans* in the presence of furfural. Both genomic data and RNA-seq data were screened for the presence of genes known to convert furfural to less toxic compounds; however, no genes were found with similarities to known furfural converting genes. This makes it likely that the beneficial effect of furfural addition is caused by increased tolerance.

Previous studies suggested that furfural toxicity is mainly caused by the formation of ROS, which are created by the large dipole moment of the aldehyde group in furfural molecules (Allen et al. 2010; Feron et al. 1991). Several genes are present in *B. coagulans* which are known to decrease the toxicity of ROS, including superoxide dismutase, thioredoxin reductase and catalase (Cabiscol et al. 2000). However, all these genes were downregulated in cultures to which furfural was added. It is therefore unlikely that these enzymes are involved in the furfural adaptation.

The presence of furfural had the largest impact on genes involved in de novo pyrimidine pathway, used for the synthesis of uracil. Uracil can also be produced from compounds present in yeast extract like cytosine. It was found that a cytosine transporter gene was strongly upregulated in precultures grown in the presence of furfural, whilst genes involved in the conversion of cytosine to uracil and cytidine were slightly upregulated. Since significant uracil and cytosine concentrations are present in yeast extract, de novo pyrimidine synthesis may not be required. The relevance of these changes in increasing furfural tolerance is unclear.

Elongation of cells was observed when grown in the presence of furfural (Fig. 2). Their length increased up to a factor 3 during precultivation and up to a factor 8 during fermentation. RNA-seq analysis showed that several pathways related to the production of cell wall compounds like peptidoglycans, bacillosamines and spermidines were found to be upregulated in the presence of furfural (Table 4). An earlier study has shown that heat stress resulted in a similar elongation of *Bacillus* cells. This also led to a shift in cell wall peptidoglycan composition. As a result, the cells showing elongation during heat stress were less vulnerable for autolysis (Novitsky et al. 1974). Another study showed that peptidoglycans and spermidine can covalently bind in cell walls, stabilizing *Bacillus* cells during osmotic stress (Wortham et al. 2007; Yokoyama et al. 1989). Apparently, elongated cell morphology and cell wall composition are related to stress response in bacilli and as such also play a role in the observed adaptation to furfural.

Sigma factors tightly regulate the expression of many genes in bacilli. One sigma factor found to be upregulated in the presence of furfural is *SigF* (Schmidt et al. 1990). *SigF* is believed to be involved in the first stage of sporulation. However, the culture used in RNA-seq analysis was in the (late) exponential growth phase, which is confirmed by high presence of *SigA*, a sigma factor only expressed during exponential growth (Qi and Doi. 1990). Expression of *SigE*, involved in the second stage of sporulation, was also not observed. It can be theorized that *SigF* may have a role apart from sporulation and may be involved in increasing cell wall strength normally required for the formation of forespores.

>Another sigma factor that was upregulated in the presence of furfural is *SigB* (van Schaik et al. 2005). Previous research showed that *SigB* expression is induced by various stress factors, varying from temperature stress to acid stress to oxygen stress (Hecker et al. 2007, Price et al. 2001). *SigB* is estimated to control the expression of up to 150 genes (Price et al. 2001). However, the function of most of these genes is unknown (Price et al. 2001). During RNA-Seq analysis, several genes were found to be upregulated which are promoted by *SigB*, such as *YsnF*, *NHaX*, *DPS/general stress factor 20U*, the *APP/OPP* transport gene cluster and *GsiC*.

According to Hecker et al. (2007), inducing a mild stress to bacilli can activate *SigB*. When *SigB* is activated, a cross-protection against different stress conditions which are otherwise lethal was observed. This cross-protection may also be triggered in *B. coagulans* cells exposed to 1 g/L of furfural during preculture. Gaidenko and Price (1998) showed that a knockout of *SigB* in *Bacillus subtilis* decreased cellular tolerance against inhibitory conditions by tenfold. It may be interesting to investigate whether upregulation of *SigB*, *YsnF* and/or *NhaX* via genetic engineering will increase the viability of *B. coagulans*, when grown directly in the presence of potentially inhibiting by-products.

## References

[CR1] Allen SA, Clark W, McCaffery JM, Cai Z, Lanctot A, Slininger PJ, Liu ZL (2010). Furfural induces reactive oxygen species accumulation and cellular damage in *Saccharomyces cerevisiae*. Biotechnol Biofuels.

[CR2] Anders S, Huber W (2010). Differential expression analysis for sequence count data. Genome Biol.

[CR3] Benjamini Y, Hochberg Y (1995). Controlling the false discovery rate: a practical and powerful approach to multiple testing. J R Stat Soc Series B Stat Methodol.

[CR4] Boisvert S, Laviolette F, Corbeil J (2010). Ray: simultaneous assembly of reads from a mix of high-throughput sequencing technologies. J Comp Biol.

[CR5] Cabiscol E, Tamarit J, Ros J (2000). Oxidative stress in bacteria and protein damage by reactive oxygen species. Int Microbiol.

[CR6] Fengel D, Wegener G (1983). Wood: chemistry, ultrastructure, reactions.

[CR7] Feron VJ, Til HP, De Vrijer F, Woutersen RA, Cassee FR, Van Bladeren PJ (1991). Aldehydes: occurrence, carcinogenic potential, mechanism of action and risk assessment. Mutat Res Gen Tox.

[CR8] Garlotta D (2001) A Literature Review of Poly(Lactic acid). J Polym Environ 9:63–84

[CR9] Gaidenko TA, Price CW (1998). General stress transcription factor ςB and sporulation transcription factor ςH each contribute to survival of *Bacillus subtilis* under extreme growth conditions. J Bacteriol.

[CR10] Gutiérrez T, Buszko ML, Ingram LO, Preston JF (2002). Reduction of furfural to furfuryl alcohol by ethanologenic strains of bacteria and its effect on ethanol production from xylose. Appl Biochem Biotechnol.

[CR11] Hecker M, Pané-Farré J, Uwe V (2007). SigB-dependent general stress response in Bacillus subtilis and related gram-positive bacteria. Annu Rev Microbiol.

[CR12] Hendriks ATWM, Zeeman G (2009). Pretreatments to enhance the digestibility of lignocellulosic biomass. Bioresource Technol.

[CR13] Koopman F, Wierckx N, de Winde JH, Ruijssenaars HJ (2010). Identification and characterization of the furfural and 5-(hydroxymethyl) furfural degradation pathways of *Cupriavidus basilensis* HMF14. Proc Natl Acad Sci.

[CR14] Laadan B, Almeida JRM, Rådström P, Hahn-Hägerdal B, Gorwa-Grauslund M (2008). Identification of an NADH-dependent 5-hydroxymethylfurfural-reducing alcohol dehydrogenase in *Saccharomyces cerevisiae*. Yeast.

[CR15] Li H (2013) Aligning sequence reads, clone sequences and assembly contigs with BWA-MEM. http://arxiv.org/abs/1303.3997 (accessed 11-2015)

[CR16] Maas RHW, Bakker RR, Jansen MLA, Visser D, de Jong E, Eggink G, Weusthuis RA (2008). Lactic acid production from lime-treated wheat straw by *Bacillus coagulans*: neutralization of acid by fed-batch addition of alkaline substrate. Appl Microbiol Biotechnol.

[CR17] Mortazavi A, Williams BA, McCue K, Schaeffer L, Wold B (2008). Mapping and quantifying mammalian transcriptomes by RNA-Seq. Nat Methods.

[CR18] Nichols NN, Sharma LN, Mowery RA, Chambliss CK, van Walsum GP, Dien BS, Iten LB (2008). Fungal metabolism of fermentation inhibitors present in corn stover dilute acid hydrolysate. Enzyme Microb Technol.

[CR19] Novitsky TJ, Chan M, Himes RH, Akagi JM (1974). Effect of temperature on the growth and cell wall chemistry of a facultative thermophilic. Bacillus J Bacteriol.

[CR20] Palmqvist E, Hahn-Hägerdal B (2000). Fermentation of lignocellulosic hydrolysates. II: inhibitors and mechanisms of inhibition. Bioresource Technol.

[CR21] Palmqvist E, Hahn-Hägerdal B (2000). Fermentation of lignocellulosic hydrolysates. II: inhibitors and mechanisms of inhibition. Bioresource Technol.

[CR22] Peng Y, Leung HC, Yiu SM, Chin FY (2012). IDBA-UD: a de novo assembler for single-cell and metagenomic sequencing data with highly uneven depth. Bioinformatics.

[CR23] Price CW, Fawcett P, Ceremonie H, Su N, Murphy CK, Youngman P (2001). Genome-wide analysis of the general stress response in *Bacillus subtilis*. Mol Microbiol.

[CR24] Qi FX, Doi RH (1990). Localization of a second SigH promoter in the *Bacillus subtilis* sigA operon and regulation of dnaE expression by the promoter. J Bacteriol.

[CR25] Schmidt R, Margolis P, Duncan L, Coppolecchia R, Moran CP, Losick R (1990). Control of developmental transcription factor sigma F by sporulation regulatory proteins SpoIIAA and SpoIIAB in *Bacillus subtilis*. Proc Natl Acad Sci.

[CR26] Seemann T (2014). Prokka: rapid prokaryotic genome annotation. Bioinformatics.

[CR27] Van der Pol EC, Bakker RR, Baets P, Eggink G (2014). By-products resulting from lignocellulose pretreatment and their inhibitory effect on fermentations for (bio)chemicals and fuels. Appl Microbiol Biotechnol.

[CR28] van der Pol EC, Bakker RR, Zeeland ANT, Sanchez Garcia D, Punt A, Eggink G (2015). Analysis of by-product formation and sugar monomerization in sugarcane bagasse pretreated at pilot plant scale: differences between autohydrolysis, alkaline and acid pretreatment. Bioresource Technol.

[CR29] van der Pol EC, Vaessen E, Weusthuis RA, Eggink G (2016). Identifying inhibitory effects of lignocellulosic by-products on growth of lactic acid producing micro-organisms using a rapid small-scale screening method. Bioresource Technol.

[CR30] van Schaik W, Tempelaars MH, Zwietering MH, de Vos WM, Abee T (2005). Analysis of the role of RsbV, RsbW, and RsbY in regulating σB activity in *Bacillus cereus*. J Bacteriol.

[CR31] Walton SL, Bisschoff KM, van Heiningen AR, van Walsum GP (2010). Production of lactic acid from hemicellulose extracts by *Bacillus coagulans* MXL-9. J Ind Microbiol Biotechnol.

[CR32] Wiegeshoff F, Beckering CL, Debarbouille M, Marahiel MA (2006). Sigma L is important for cold shock adaptation of *Bacillus subtilis*. J Bacteriol.

[CR33] Wortham BW, Oliveira MA, Patel CH (2007). Polyamines in Bacteria: pleiotropic effects yet specific mechanisms. Adv Exp Med Biol.

[CR34] Yokoyama K, Mizuguchi H, Araki Y, Kaya S, Ito E (1989). Biosynthesis of linkage units for teichoic acids in gram-positive bacteria: distribution of related enzymes and their specificities for UDP-sugars and lipid-linked intermediates. J Bacteriol.

